# A quantitative study of spinothalamic neurons in laminae I, III, and IV in lumbar and cervical segments of the rat spinal cord

**DOI:** 10.1002/cne.21811

**Published:** 2008-11-01

**Authors:** Khulood M Al-Khater, Robert Kerr, Andrew J Todd

**Affiliations:** Spinal Cord Group, Institute of Biomedical & Life Sciences, University of GlasgowGlasgow, G12 8QQ, United Kingdom

**Keywords:** posterior triangular nucleus of thalamus, neurokinin 1 receptor, dorsal horn, confocal microscopy

## Abstract

The major ascending outputs from superficial spinal dorsal horn consist of projection neurons in lamina I, together with neurons in laminae III–IV that express the neurokinin 1 receptor (NK1r) and have dendrites that enter the superficial laminae. Some neurons in each of these populations belong to the spinothalamic tract, which conveys nociceptive information via the thalamus to cortical areas involved in pain. A projection from the cervical superficial dorsal horn to the posterior triangular nucleus (PoT) has recently been identified. PoT is at the caudal end of the thalamus and was not included in injection sites in many previous retrograde tracing studies. We have injected various tracers (cholera toxin B subunit, Fluoro-Gold, and fluorescent latex microspheres) into the thalamus to estimate the number of spinothalamic neurons in each of these two populations, and to investigate their projection targets. Most lamina I and lamina III/IV NK1r-immunoreactive spinothalamic neurons in cervical and lumbar segments could be labeled from injections centered on PoT. Our results suggest that there are 90 lamina I spinothalamic neurons per side in C7 and 15 in L4 and that some of those in C7 only project to PoT. We found that 85% of the lamina III/IV NK1r-immunoreactive neurons in C6 and 17% of those in L5 belong to the spinothalamic tract, and these apparently project exclusively to the caudal thalamus, including PoT. Because PoT projects to second somatosensory and insular cortices, our results suggest that these are major targets for information conveyed by both these populations of spinothalamic neurons.

The spinal dorsal horn receives a major input from primary afferent axons. This input is organized according to modality, with nociceptive afferents terminating mainly in laminae I and II (Rexed, 1954; Todd and Koerber, 2005). Although the axons of most dorsal horn neurons remain within the spinal cord, some of these cells (projection neurons) have axons that terminate in the brain, and these form an important output from the region. Projection neurons are not uniformly distributed within the dorsal horn, but are concentrated in lamina I and scattered throughout the deeper laminae (III–VI).

The majority of lamina I projection neurons in the rat express the neurokinin 1 receptor (NK1r) on which substance P acts (Ding et al., 1995; Li et al., 1996, 1998; Marshall et al., 1996; Todd et al., 2000; Spike et al., 2003). Another population of projection neurons consists of large NK1r-immunoreactive cells with somata in laminae III or IV and long dorsal dendrites that reach lamina I (Naim et al., 1997; Todd et al., 2000). Ablation of NK1r-expressing neurons in the superficial dorsal horn results in loss of hyperalgesia in chronic pain models (Mantyh et al., 1997; Nichols et al., 1999), which suggests that projection neurons with the NK1r may play an important role in the development of hyperalgesia. The supraspinal projection targets of these two groups of cells have been studied most extensively in the rat and include the caudal ventrolateral medulla (CVLM), the lateral parabrachial area (LPb), the periaqueductal gray matter (PAG), and the thalamus (Menétrey et al., 1982,^1983^; Cechetto et al., 1985; Hylden et al., 1989; Lima and Coimbra, 1988;^1989^; Burstein et al., 1990; Lima et al., 1991; Ding et al., 1995; Todd et al., 2000; Spike et al., 2003; Gauriau and Bernard, 2004a).

These projections are predominantly contralateral, although a significant number of cells appear to project bilaterally (Spike et al., 2003). Both lamina I and lamina III–IV projection neurons that possess the NK1r are densely innervated by substance P-containing primary afferent axons (Naim et al., 1997; Todd et al., 2002) and thus provide a route through which these afferents, which are known to be nociceptors (Lawson et al., 1997), can activate brain regions involved in pain mechanisms.

Projections from lamina I neurons to the thalamus have been demonstrated in primates (Trevino and Carstens, 1975; Willis et al., 1979; Apkarian and Hodge, 1989; Zhang and Craig, 1997), cats (Trevino and Carstens, 1975; Jones et al., 1987; Craig et al., 1989; Zhang et al., 1996; Mouton and Holstege, 1998; Klop et al., 2004,2005a), and rats (Kevetter and Willis, 1983; Lima and Coimbra, 1988; Burstein et al., 1990; Li et al., 1996; Marshall et al., 1996; Kobayashi, 1998; Kayalioglu et al., 1999; Yu et al., 2005), and several studies have attempted to determine the number of lamina I spinothalamic tract neurons in these species (Lima and Coimbra, 1988; Apkarian and Hodge, 1989; Burstein et al., 1990; Klop et al., 2004,2005a; Yu et al., 2005). Recently, Gauriau and Bernard (2004a) have identified a substantial projection from the superficial dorsal horn of the rat cervical spinal cord to the triangular part of the posterior thalamic nuclear group (PoT), an area that projects to the secondary somatosensory (S2) and insular cortices (Shi and Cassell, 1998; Linke and Schwegler, 2000; Gauriau and Bernard, 2004b). The PoT lies at the extreme caudal end of the thalamus and was apparently not included in many previous retrograde tracing studies.

It has been shown that some of the lamina III/IV NK1r-immunoreactive neurons in rat lumbar spinal cord project to the thalamus (Marshall et al., 1996; Naim et al., 1997). However, the proportion of those in lumbar or cervical cord that belong to the spinothalamic tract is not known.

In the present study we have re-examined the spinothalamic projection from lamina I and from the NK1r-expressing lamina III/IV cells of lumbar and cervical segments of the rat spinal cord. Our main aim was to address the following questions: 1) has the number of spinothalamic neurons in lamina I been underestimated in previous studies, due to failure to fill the PoT?; 2) is there a projection from lumbar segments to PoT?; and 3) what proportion of lamina III/IV NK1r-expressing cells in lumbar and cervical cord belong to the spinothalamic tract and where do their axons terminate within the thalamus?

A recent study by Yu et al. (2005) reported that spinothalamic neurons made up 9% of the neuronal population in lamina I of the rat cervical cord. However, we have provided evidence that only ∼5% of lamina I neurons in lumbar cord project to the brain (Spike et al., 2003), and it has been shown that in the cat, spinothalamic lamina I cells are greatly outnumbered by other projection neurons (Mouton and Holstege, 1998; Klop et al., 2005b). Therefore an additional aim of this study was to determine the proportion of lamina I cells in cervical cord that belong to the spinothalamic tract.

## MATERIALS AND METHODS

### Surgery

All experiments were approved by the Ethical Review Process Applications Panel of the University of Glasgow and were performed in accordance with the UK Animals (Scientific Procedures) Act 1986.

Twenty-five adult male Wistar rats (240–330 g; Harlan, Loughborough, UK) were anesthetized either with ketamine and xylazine (73.3 and 7.3 mg/kg i.p., respectively, supplemented as necessary) or with a volatile anesthetic (halothane or isofluorane). They were then placed in a stereotaxic frame and in those cases in which halothane or isofluorane were used, the anesthetic was administered through a mask attached to the frame. Each rat received injections of one or more of the following tracers into the left thalamus: 1% cholera toxin B subunit (CTb; Sigma, Poole, UK), 4% Fluoro-Gold (Fluorochrome, Englewood, CO), or rhodamine-labeled fluorescent latex microspheres (undiluted, Lumafluor, Naples, FL). All injections were made through glass micropipettes, and these were left in place for 5 minutes after the completion of each injection to minimize leakage of tracer back up the track. In those cases in which two tracers were injected, a different pipette was used for each tracer. Four different injection strategies were used (Table 1):

1. Injection of Fluoro-Gold into thalamic regions known to receive projections from superficial dorsal horn neurons, including PoT (experiments Thal1–5).
2. Injection of CTb or Fluoro-Gold targeted on the PoT (PoT1–10).
3. Injection of CTb into PoT and of Fluoro-Gold into other thalamic areas known to receive projections from superficial dorsal horn neurons (double injection, DI1–7).
4. Injection of fluorescent latex microspheres into PoT (FLM1–3).

**Table 1:** Details of Tracer Injections for Each Experiment^1^

	Tracer (in nl)		
	
Experiment	CTb	FG	FLM
Thal1		100 × 5	
Thal2		100 × 6	
Thal3		100 × 6	
Thal4		100 × 6	
Thal5		100 × 5	
PoT1	250		
PoT2		100	
PoT3		100 × 2	
PoT4		100 × 2	
PoT5		100	
PoT6		50	
PoT7	200		
PoT8	200		
PoT9	50		
PoT10	50		
DI1	200	100 × 3	
DI2	200	100 × 3	
DI3	200	100 × 3	
DI4	200	100 × 3	
DI5	200	100 × 6	
DI6	200	100 × 6	
DI7	200	100 × 6	
FLM1			50
FLM2			50 × 2
FLM3			50 × 2

1 The approximate volume of tracer for each injection is shown. When a tracer was injected at more than one site this is also indicated. CTb, cholera toxin b; FG, Fluoro-Gold; FLM, fluorescent latex microspheres.

Details of the tracer(s) used and volumes injected in each experiment are given in Table 1. The experiments with fluorescent latex microspheres were performed because these show very little spread (resulting in restricted injection sites) and are not taken up by undamaged fibers (Katz et al., 1984).

All animals made an uneventful recovery from anesthesia. After a survival period of 3 days they were re-anesthetized with pentobarbitone (300 mg i.p.) and perfused through the heart with a fixative that contained 4% freshly depolymerized formaldehyde. Lumbar and cervical spinal cord segments were removed and stored in fixative for 24 hours, whereas the brain was cryoprotected in 30% sucrose in fixative overnight. In order to measure the length of the C7 segment, this was photographed in situ in three experiments (DI5 and 6 and FLM3), and the distance between the most rostral and caudal rootlets of the C7 dorsal roots was determined.

### Tissue processing and immunocytochemistry

The brain region surrounding the injection site was cut into 100-μm-thick coronal sections with a freezing microtome. Brains from animals that had received injections of CTb (with or without Fluoro-Gold) were cut into five complete series, and sections from at least one of these series were reacted with goat anti-CTb (List Biological Laboratories, Campbell, CA; diluted 1:50,000) by using an immunoperoxidase method as described previously (Spike et al., 2003). All remaining sections from animals that had received injections of Fluoro-Gold in addition to CTb were mounted with anti-fade medium (Vectashield, Vector, Peterborough, UK) for detection of Fluoro-Gold with epifluorescent illumination and an UV filter set. All sections through the injection sites from animals that had received only Fluoro-Gold or fluorescent latex microspheres were mounted in serial order. Those from Fluoro-Gold-injected rats were mounted with Vectashield and viewed with UV epifluorescence. Sections from animals that received injections of fluorescent latex microspheres were mounted in Gel-Mount medium (Sigma-Aldrich, Poole, Dorset, UK) because the microspheres are destroyed by glycerol, and these were viewed with bright- and darkfield illumination. In all cases the spread of tracer from the injection sites was plotted onto drawings of the thalamus (Paxinos and Watson, 2005). Representative examples were photographed.

The C7 and L4 spinal cord segments from all animals, apart from those that received injections of fluorescent latex microspheres, were initially notched on the left side, so that the two sides could subsequently be distinguished, and were then cut into 60-μm-thick transverse sections with a Vibratome. Some or all of these sections were reacted to reveal neuronal nuclei, the tracer(s), and in some cases the NK1r. Sections were incubated free-floating at 4°C for 3 days in one of the following antibody combinations:

1. Mouse monoclonal antibody NeuN (against a neuronal nuclear protein, Millipore, Watford, UK; diluted 1:1,000), goat anti-CTb (1:5,000), and rabbit anti-NK1r (Sigma-Aldrich; 1:10,000) (experiments PoT1 and PoT7–10).
2. NeuN, guinea-pig anti-Fluoro-Gold (Protos Biotech, New York, NY; 1:500) and rabbit anti-NK1r (experiments Thal2–5 and PoT4–6).
3. NeuN, goat anti-CTb, and guinea-pig anti-Fluoro-Gold (experiments DI1–7).

The sections were then incubated overnight at 4°C in species-specific secondary antibodies that were raised in donkey and conjugated to either Alexa 488 (Invitrogen, Paisley, UK; 1:500), or to Rhodamine Red or Cy5 (Jackson ImmunoResearch, West Grove, PA; 1:100). Sections from experiments Thal1 and PoT2–3 were incubated for 3 days in rabbit anti-Fluoro-Gold (Millipore, diluted 1:5,000), overnight in the Fab′ fragment of goat anti-rabbit IgG conjugated to rhodamine (Jackson ImmunoResearch), and then for 2 days in rabbit anti-NK1r, followed by 1 day in donkey anti-rabbit IgG conjugated to Alexa 488. Following immunostaining, some of the sections from the C7 segment of experiments Thal2–5 were incubated for 30 minutes in the fluorescent nuclear stain 4′,6-diamidino- 2-phenylindole (DAPI; Sigma-Aldrich; 1 μg/ml in phosphate-buffered saline). All sections were mounted in Vectashield and stored at −20°C.

The right sides of the C6 and L5 segments (contralateral to the injection sites) from all experiments were notched to allow identification of the caudal end, and cut into 60-μm-thick parasagittal sections (50 μm thick for experiments FLM2–3). All of these sections were reacted to reveal NK1r, CTb, and/or Fluoro-Gold (when these had been injected), and in some cases, NeuN. Sections were incubated for 3 days at 4°C in one of the following antibody combinations:

1. Rabbit anti-NK1r and goat anti-CTb (experiments PoT1 and PoT7–10).
2. Rabbit anti-NK1r and guinea-pig anti-Fluoro-Gold (experiments Thal2–5 and PoT4–6).
3. Rabbit anti-NK1r, goat anti-CTb, and guinea-pig anti-Fluoro-Gold (experiments DI1–7).
4. Rabbit anti-NK1r and NeuN (FLM1-3).

The sections were then incubated overnight at 4°C in species-specific secondary antibodies that were raised in donkey and conjugated to Alexa 488, Rhodamine Red, or Cy5. Sections from experiments Thal1 and PoT2–3 were processed sequentially in rabbit antibodies against Fluoro-Gold and NK1r, as described above. Sections were mounted in serial order in Vectashield or Gel-Mount and stored at −20°C.

### Antibody characterization

The NK1r antibody (catalogue number S8305) was raised in rabbit against a peptide corresponding to amino acids 393–407 at the C-terminus of the rat NK1 receptor, which was conjugated to keyhole limpet hemocyanin. The antibody recognizes a 46-kDa band in Western blots of rat brain extracts, and this staining is specifically abolished by preabsorption of the antibody with the immunizing peptide (manufacturer's specification). It has been shown that there is no immunostaining with this antibody in sections of medulla and cervical spinal cord from mice in which the NK1r has been deleted (NK1−/−), whereas staining is present in sections from wild-type mice (Ptak et al., 2002).

The mouse monoclonal antibody NeuN (catalogue number MAB377) was generated against cell nuclei extracted from mouse brain and was found to react with a protein specific to neurons (Mullen et al., 1992). We have shown that this antibody apparently labels all neurons (and no glial cells) within the rat spinal cord (Todd et al., 1998).

The goat polyclonal antibody (catalogue number 703) was raised against CTb, whereas rabbit (catalogue number AB153) and guinea-pig (catalogue number NM101) antibodies were raised against Fluoro-Gold. Specificity of each of these tracer antibodies was shown by the lack of staining in regions of the CNS that did not contain neurons that had taken up and transported the tracer and by the presence of immunostaining in populations of neurons that are known to project to the injection sites. In addition, the specificity of the Fluoro-Gold antibodies was directly confirmed by comparing Fluoro-Gold fluorescence (observed with an UV filter set) with that for anti-Fluoro-Gold in individual neurons. In all cases examined, there was a perfect match between the two types of fluorescence.

### Confocal microscopy and analysis

Transverse sections from the rats that had received injections of CTb and/or Fluoro-Gold were used to analyze the number of retrogradely labeled neurons in lamina I in each experiment. From each animal, 10 sections from the C7 segment and 20 sections from the L4 segment were randomly selected and viewed with epifluorescence. All lamina I neurons on the right side of each section that were labeled with CTb and/or Fluoro-Gold were identified and then scanned with a confocal microscope (Bio-Rad MRC1024 or Radiance 2100; Bio-Rad, Hemel Hempstead, UK) through dry (4×, 10×, 20×) and oil-immersion (60×) lenses. In order to correct for the overcounting that results from the presence of transected cells at the section surfaces, cells were only included in the sample if their nucleus (identified as a filling defect) was entirely contained within the Vibratome section, or if part of the nucleus was present in the first optical section in the z-series (corresponding to the top of the Vibratome section); they were excluded if part of the nucleus was present in the last optical section in the z-series (Spike et al., 2003). The low-magnification images were used to plot the position of the cells on an outline of the dorsal horn. In this way the mean number of retrogradely labeled lamina I cells containing CTb, Fluoro-Gold, or both tracers per 600 μm (C7) or 1,200 μm (L4) was determined for each experiment. Darkfield microscopy was used to distinguish laminar boundaries (see Results), and retrogradely labeled cells were judged to be in lamina I if they were very close to the dorsal border of the dorsal horn or lay dorsal to the dark band identified as lamina II with darkfield microscopy. For most of the experiments in which a single tracer was used (Thal2–5, PoT1, PoT4–10), the presence or absence of NK1r was also noted for each retrogradely labeled lamina I cell.

In order to determine the proportion of lamina I neurons that belonged to the spinothalamic tract in the C7 segment, six Vibratome sections that had been immunostained and incubated in DAPI were randomly selected from four experiments in which extensive injections of Fluoro-Gold had been made (Thal2–5). These were scanned through a 40× oil-immersion lens to reveal NeuN, NK1r, Fluoro-Gold, and DAPI. In each case, z-series consisting of 16 optical sections at 1 μm spacing were obtained from the entire mediolateral extent of lamina I on the right side of the spinal cord. To determine the degree of tissue shrinkage, the thickness of each section was measured by scanning top and bottom surfaces at three different locations and calculating the mean distance between these surfaces (Polgár et al., 2004). Drawings of these sections were made with Neurolucida for Confocal (MicroBrightField, Colchester, VT), and the boundaries of lamina I were identified, based on the pattern of NK1r immunostaining (see Results). A modification of the optical disector method (Sterio, 1984) was then used to determine the total number of neurons in a 9-μm-thick slice through lamina I, by examining all optical sections and counting all of the neurons with nuclei that had a bottom surface between the 3rd and 11th optical sections in the z-series (Todd et al., 1998; Spike et al., 2003). To avoid bias, the selection of neurons was made before the Fluoro-Gold immunoreactivity was viewed. For each neuron that was included in the disector sample, the presence or absence of Fluoro-Gold was then noted. The proportion of lamina I neurons that belonged to the spinothalamic tract was estimated in two different ways.

First, for each rat the number of retrogradely labeled neurons in the disector samples was divided by the total number of lamina I neurons in the samples, and this value was averaged for the four rats. Second, the disector samples were used to estimate the total number of lamina I neurons per 600 μm in the C7 segment, and this was subsequently compared with the numbers of retrogradely labeled lamina I neurons found in this segment in the experiments in which CTb and/or Fluoro-Gold were injected. In order to estimate the total number of lamina I neurons in this way, we used the following formula to correct for tissue shrinkage (Polgár et al., 2004):

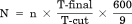

where N is the number of lamina I neurons per 600 μm of spinal cord, n is the number counted in the 9-μm disector, T-final is the thickness of the particular section measured after any shrinkage, and T-cut is the original section thickness cut (60 μm).

For each of the rats that had received injections of CTb and/or Fluoro-Gold, the entire series of parasagittal sections through the gray matter of the right side of both the C6 and L5 segments were used to count the number of NK1 receptor-immunoreactive neurons that had cell bodies in laminae III or IV and dorsal dendrites that could be followed into laminae I or II, and to determine the proportion of these cells that was retrogradely labeled. Darkfield microscopy was used to ensure that all cells had their somata ventral to lamina II. Sections were initially viewed with epifluorescence through a 20× lens to identify NK1 receptor-immunoreactive cell bodies in laminae III or IV. In most cases it was possible to determine with epifluorescence whether the dendrites of these cells entered the superficial dorsal horn (laminae I or II). However, in some cases (particularly when dendrites had to be followed into serial sections) it was necessary to scan with the confocal microscope. Confocal microscopy was also used to determine whether the cells were retrogradely labeled with CTb and/or Fluoro-Gold, to measure the distance between the cell body and the overlying dorsal white matter, and to ensure that cell bodies that were cut by the Vibratome and appeared on the adjacent surfaces of serial sections were not counted twice. During the course of the study (see Results), we observed that retrogradely labeled lamina III/IV NK1r-immunoreactive neurons in the L5 segment appeared to be more common in the medial part of the dorsal horn than in the lateral part. We investigated this by assigning all of the NK1r-immunoreactive cells in this segment with dorsal dendrites that entered the superficial dorsal horn into one of two groups, medial and lateral, based on the section in which they were present and, if necessary, their (mediolateral) depth within that section. In experiments in which the number of these cells was even, the two groups were of equal size, whereas in the other experiments the extra cell was included in the lateral group.

For the three rats that had received injections of fluorescent latex microspheres (FLM1–3), complete series of parasagittal sections through the right side of the C6 and L5 segments were examined with epifluorescence through a 40× oil-immersion lens. All retrogradely labeled neurons in lamina I, as well as retrogradely labeled neurons in laminae III and IV that were NK1r-immunoreactive, were identified. Care was taken to avoid double-counting by ensuring that cells at the cut surface of a section were not counted on the adjacent section. For the retrogradely labeled lamina III/IV cells we determined whether dorsal dendrites could be followed into the superficial laminae (I–II) in each case.

Figures were composed with Adobe Photoshop (version 7.0). In some cases, image brightness and contrast were adjusted by using the levels setting.

## RESULTS

### Injection sites for CTb and Fluoro-Gold experiments

**Figure 1:**
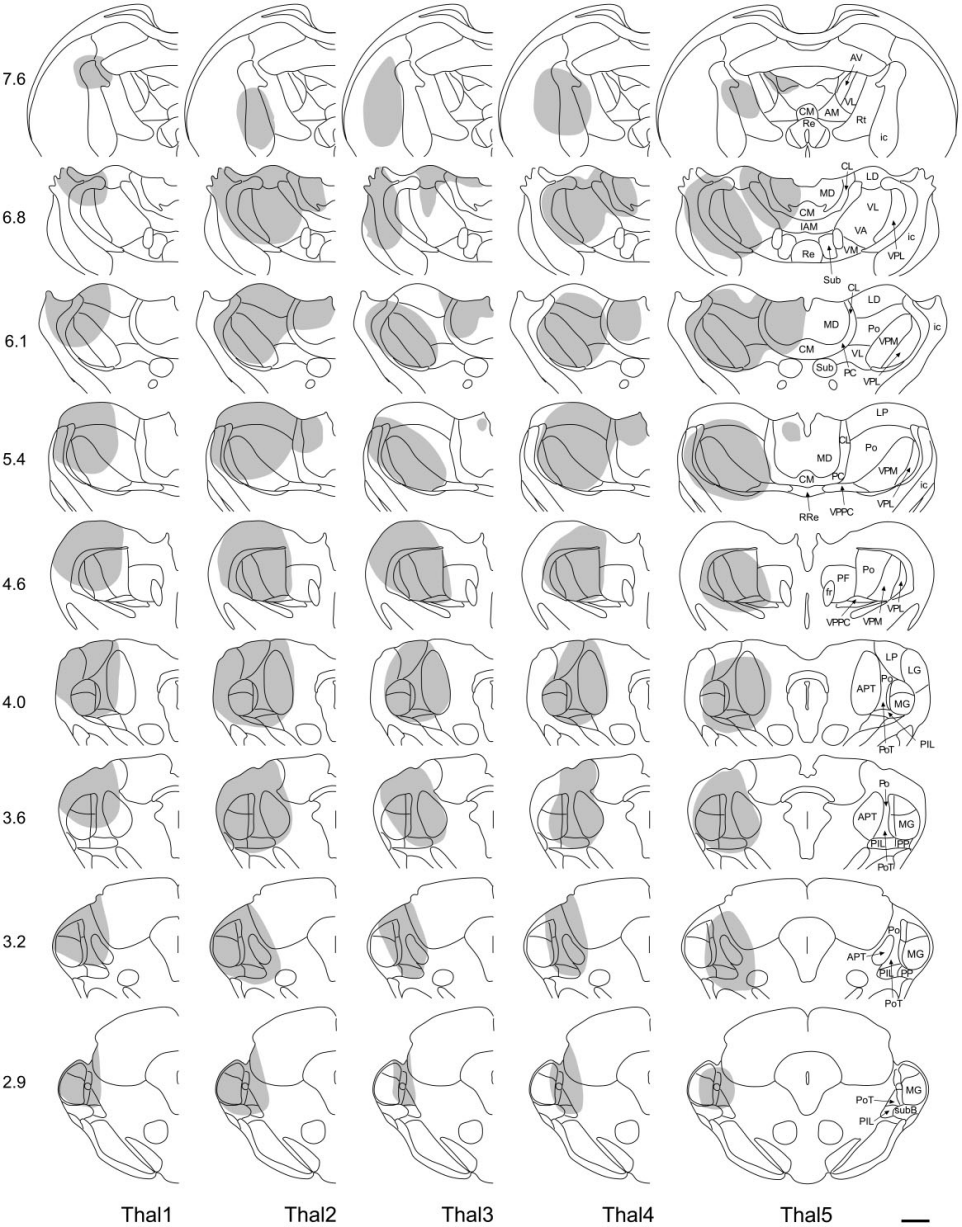
Injection sites for experiments Thal1–Thal5. Drawings show the spread of Fluoro-Gold (shaded area) in these experiments. Each vertical column represents a single experiment, and the experiment number (corresponding to those in Tables 1, 2, and 5) is shown at the bottom of the column. Numbers at the top left of each drawing give the approximate position of the section anterior to the interaural plane. Drawings are based on those in Paxinos and Watson (2005). AM, anteromedial thalamic nucleus; APT, anterior pretectal nucleus; AV, anteroventral thalamic nucleus; CL, centrolateral thalamic nucleus; CM, central medial thalamic nucleus; fr, fasciculus retroflexus; IAM, interanteromedial thalamic nucleus; ic, internal capsule; LD, laterodorsal thalamic nucleus; LG, lateral geniculate nucleus; LP, lateral posterior thalamic nucleus; MD, mediodorsal thalamic nucleus; MG, medial geniculate nucleus; PC, paracentral thalamic nucleus; PF, parafascicular thalamic nucleus; PIL, posterior intralaminar thalamic nucleus; Po, posterior thalamic nuclear group; PoT, posterior thalamic nuclear group, triangular part; PP, peripeduncular nucleus; Re, reuniens thalamic nucleus; RRE, retrouniens area; Rt, reticular thalamic nucleus; Sub, submedius thalamic nucleus; SubB, subbrachial nucleus; VA, ventral anterior thalamic nucleus; VL, ventrolateral thalamic nucleus; VM, ventromedial thalamic nucleus VPL, ventral posterolateral thalamic nucleus; VPM, ventral posteromedial thalamic nucleus; VPPC, ventral posterior thalamic nucleus, parvicellular part. Scale bar = 1 mm (also applies to Figs. 2 and 3).

**Figure 2:**
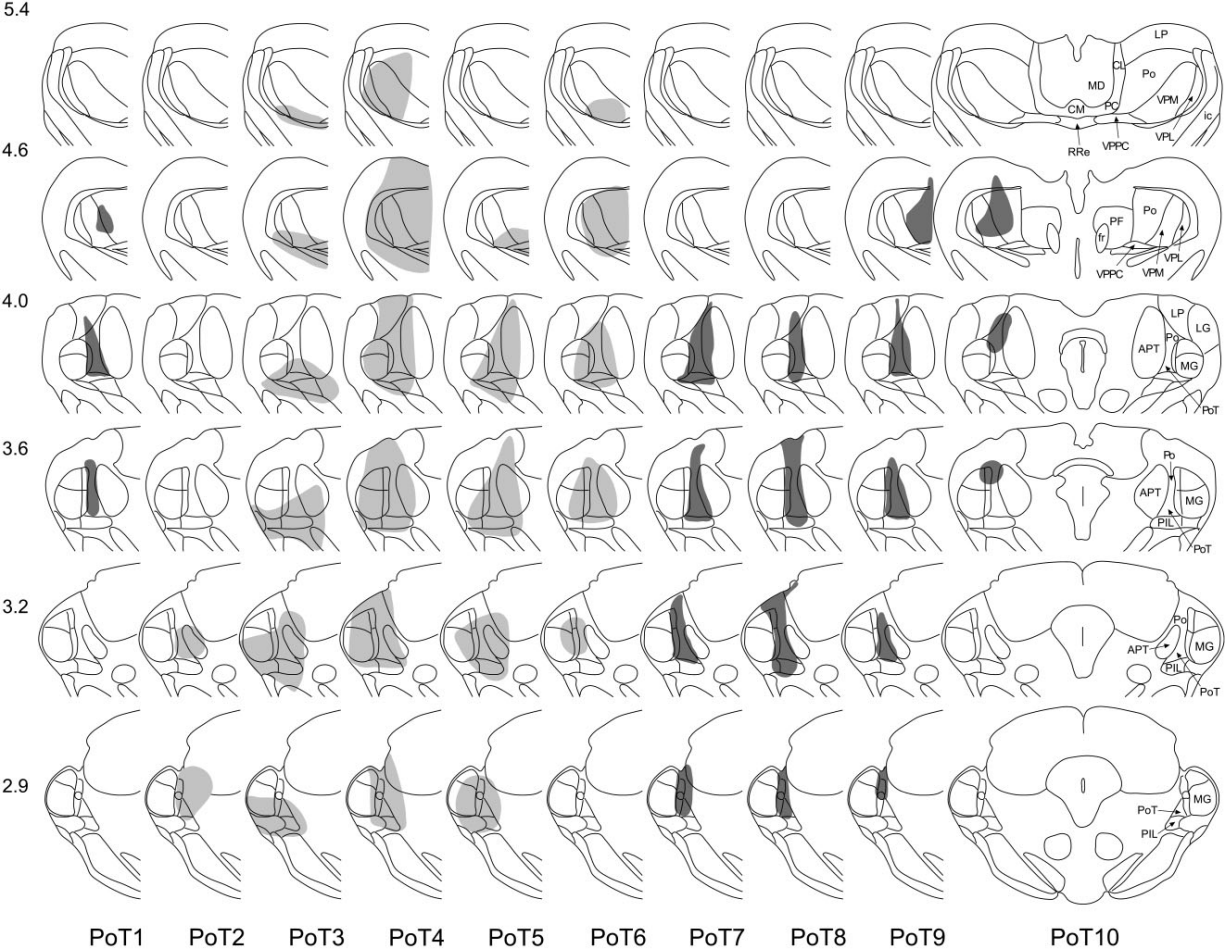
Injection sites for experiments PoT1–PoT10. Drawings show the spread of CTb (dark shading) or Fluoro-Gold (light shading) in these experiments. Each column represents a single experiment, and the experiment number (corresponding to those in Tables 1, 3, and 6) is shown at the bottom of the column. Numbers at the top left of each drawing give the approximate position of the section anterior to the interaural plane. Drawings are based on those in Paxinos and Watson (2005). Abbreviations as in Figure 1.

**Figure 3:**
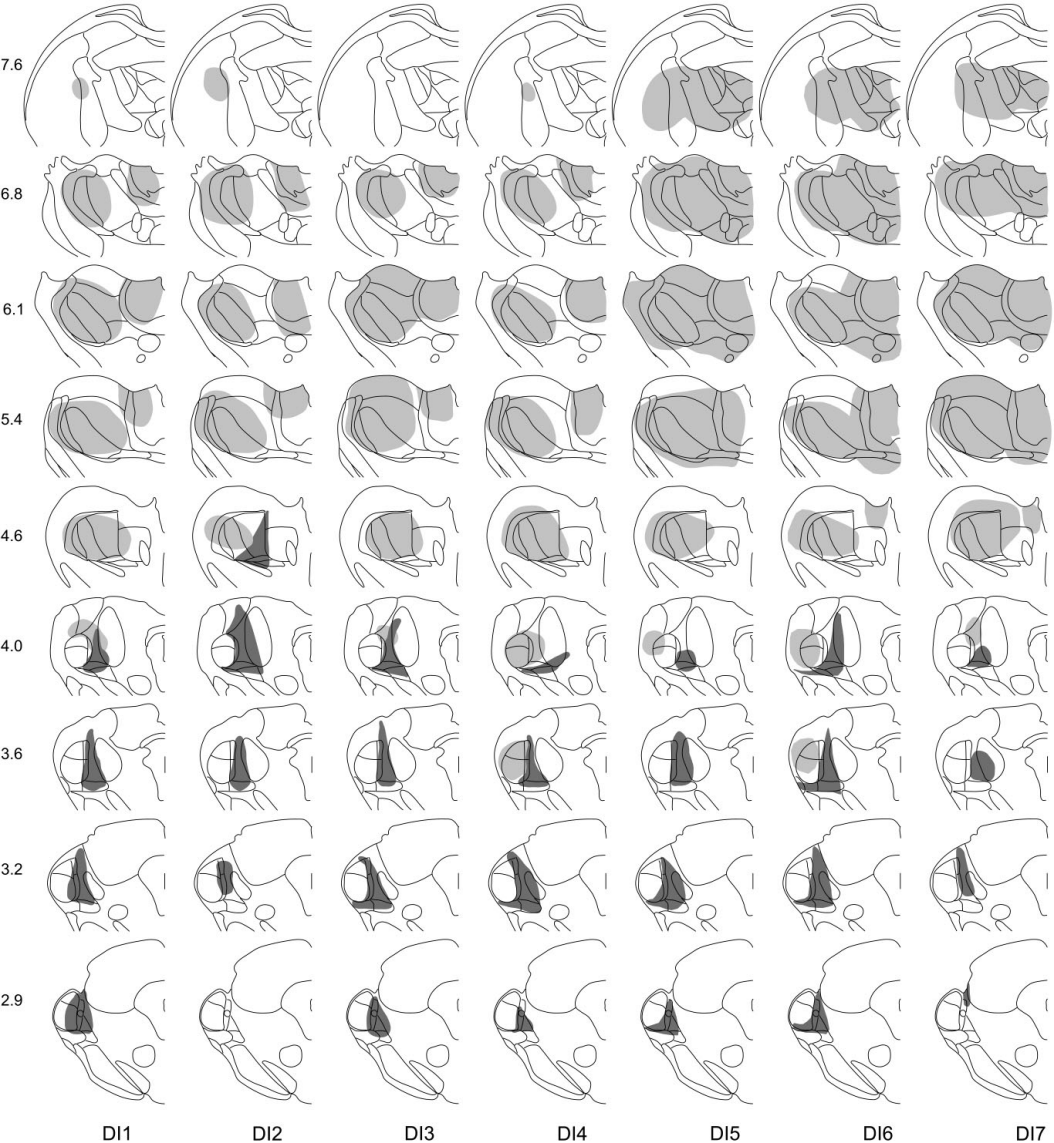
Injection sites for experiments DI1–DI7. Drawings show the spread of Fluoro-Gold (light shading) and CTb (dark shading) in these experiments. Each vertical column represents a single experiment, and the experiment number (corresponding to those in Tables 1, 4, and 7) is shown at the bottom of the column. Numbers at the top left of each drawing give the approximate position of the section anterior to the interaural plane. Drawings are based on those in Paxinos and Watson (2005). For labeling of structures shown within these drawings, see Figure 1.

**Figure 4:**
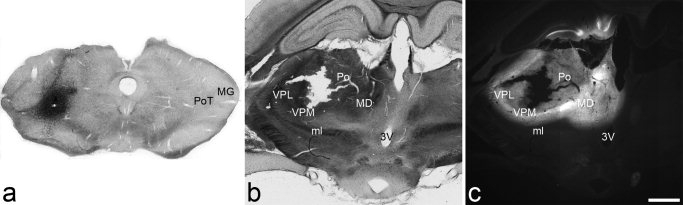
Examples of CTb and Fluoro-Gold injection sites. **a:** A section (interaural ∼3.8 mm) through an injection of CTb that was targeted on the PoT nucleus (experiment DI1). The section was reacted with an immunoperoxidase method to reveal CTb. **b, c:** Brightfield and fluorescence micrographs of a section (interaural ∼5.2 mm) through part of a Fluoro-Gold injection site (experiment DI7). Note the necrotic center and the spread of Fluoro-Gold fluorescence from this region. 3V, 3rd ventricle; ml, medial lemniscus; other abbreviations as in Figure 1. Scale bar = 1 mm in c (applies to all).

The spread of tracer within the thalamus in each of these experiments is illustrated in Figures 1–3, and representative photomicrographs of injection sites are shown in Figure 4.

**Table 2:** Quantitative Data for Retrogradely Labelled Lamina I Neurons in Thal Experiments^1^

Experiment	C7 (cells per 600 μm)	L4 (cells per 1,200 μm)
Thal1	25	8
Thal2	22	3
Thal3	23	5
Thal4	24	9
Thal5	22	10
Mean	23.2	7

1 Counts of retrogradely labeled lamina I cells in the experiments in which Fluoro-Gold injections were targeted at all thalamic regions known to receive projections from superficial dorsal horn neurons. In each experiment, cells were counted in 10 randomly selected transverse 60-μm sections from the C7 segment and in 20 such sections from the L4 segment.

**Table 3:** Quantitative Data for Retrogradely Labeled Lamina I Neurons in PoT Experiments^1^

Experiment	C7 (cells per 600 μm)	L4 (cells per 1,200 μm)
PoT1	20	5
PoT2	18	5
PoT3	18	6
PoT4	36	6
PoT5	26	
PoT6	26	9
PoT7	24	10
PoT8	25	9
PoT9	28	7
PoT10	18	4
Mean^2^	24.6	7.1

1 Counts of retrogradely labeled lamina I cells in the experiments in which tracer injections were targeted on PoT. In each experiment, cells were counted in 10 randomly selected transverse 60-μm sections from the C7 segment and in 20 such sections from the L4 segment. No results were obtained from the L4 segment in experiment PoT5.

2 In experiment PoT10 the tracer did not spread into PoT and therefore these values were not included in the calculation of the means.

**Table 4:** Quantitative Data for Retrogradely Labeled Lamina I Neurons in DI Experiments^1^

	C7 (cells per 600 μm)					L4 (cells per 1,200 μm)				
		
Experiment	FG only	CTb only	FG+CTb	% with CTb^2^	Total	FG only	CTb only	FG+CTb	% with CTb^2^	Total
DI1	1	9	19	97	29	1	6	3	90	10
DI2	0	14	7	100	21	1	4	5	90	10
DI3	0	17	5	100	22	1	6	2	89	9
DI4	6	2	8	63	16	3	1	7	73	11
DI5	4	5	12	81	21	1	1	2	75	4
DI6	16	8	4	43	28	6	4	1	45	11
DI7	4	1	15	80	20	6	2	2	40	10
mean	4.4	8	10	81	22.4	2.7	3.4	3.1	72	9.3

1 Counts of retrogradely labeled lamina I cells in the experiments in which cholera toxin B (CTb) was targeted at PoT and Fluoro-Gold (FG) at other thalamic regions known to receive projections from superficial dorsal horn neurons. In each experiment, cells were counted in 10 randomly selected transverse 60-μm sections from the C7 segment and in 20 such sections from the L4 segment.

2 These columns shows the percentage of all retrogradely labeled cells that contained CTb.

**Table 5:** Quantitative Data for Lamina III/IV NK1r Cells From Thal Experiments^1^

	C6			L5				
		
Experiment	No.	STT	%	No.	STT	%	Med %	Lat %
Thal1	13	12	92	27	7	26	38	14
Thal2	15	15	100	24	0	0	0	0
Thal3	12	10	83	28	7	25	43	7
Thal4	21	20	95	23	5	22	18	25
Thal5	16	15	94	28	3	11	21	0
Mean	15.4	14.4	93	26	4.4	17	24	9

1 Counts of the lamina III/IV NK1r cells with dendrites that could be followed into the superficial dorsal horn in complete series of parasagittal sections of C6 and L5. For each segment, the total number of cells of this type (No.), and the number (STT) and percentage (%) that were retrogradely labeled, are given. For the L5 segment, the percentage of cells of this type in the medial (Med) or lateral (Lat) group that were retrogradely labeled is also provided (see text for further details).

**Table 6:** Quantitative Data for Lamina III/IV NK1r Cells From PoT Experiments^1^

	C6			L5				
		
Experiment	No.	STT	%	No.	STT	%	Med %	Lat %
PoT3	15	14	93	15	4	27	43	13
PoT4	18	14	78	18	3	18	33	0
PoT5	15	15	100	15	2	13	29	0
PoT6	11	9	82	26	4	15	31	0
PoT7	22	20	91	24	4	17	17	17
PoT8	13	9	69	23	7	30	36	25
PoT9	14	12	86	16	4	25	50	0
PoT10	21	8	38	24	2	8	8	8
Mean^2^	15.4	13.3	86	19.6	4	21	34	8

1 Counts of the lamina III/IV NK1r cells with dendrites that could be followed into the superficial dorsal horn in complete series of parasagittal sections of C6 and L5. For each segment, the total number of cells of this type (No.), and the number (STT) and percentage (%) that were retrogradely labeled, are given. For the L5 segment, the percentage of cells of this type in the medial (Med) or lateral (Lat) group that were retrogradely labeled is also provided (see text for further details).

2 The mean value excludes the results for PoT10, since in this experiment there was no spread of tracer into the PoT.

**Table 7:** Quantitative Data for Lamina III/IV NK1r Cells From DI Experiments^1^

	C6						L5							
		
Experiment	No.	STT	CTb	FG	DL	%	No.	STT	CTb	FG	DL	%	Med %	Lat %
DI1	12	10	5	0	5	83	16	2	0	0	2	13	25	0
DI2	19	16	16	0	0	84	27	0	0	0	0	0	0	0
DI3	20	16	16	0	0	80	24	3	3	0	0	13	17	8
DI4	16	15	6	3	6	94	15	2	1	0	1	13	29	0
DI5	13	9	8	0	1	69								
DI6	20	16	16	0	0	80	25	6	5	0	1	24	33	15
DI7	10	6	6	0	0	60	21	2	2	0	0	10	20	0
Mean	15.7	12.6	10.4	0.4	1.7	79	21.3	2.5	1.8	0	0.7	12	21	4

1 Counts of the lamina III/IV NK1r cells with dendrites that could be followed into the superficial dorsal horn in complete series of parasagittal sections of C6 and L5. For each segment, the total number of cells of this type (number), and the number (STT) and percentage (%) that were retrogradely labeled with either tracer, are given. The retrogradely labeled cells are further divided into those that were labeled with cholera toxin B (CTb) only (CTb), Fluoro-Gold only (FG), or both tracers (double-labeled [DL]). For the L5 segment, the percentage of cells of this type in the medial (Med) or lateral (Lat) group that were retrogradely labeled with either tracer is also provided (see text for further details). Note that no data were available for the L5 segment in experiment DI5.

Injections of Fluoro-Gold aimed at thalamic regions known to receive projections from the superficial dorsal horn (Thal1–5) were successful in all cases (Fig. 1). The injection sites included PoT, the posterior thalamic nuclear group (Po), and the ventrobasal (VB) complex (ventral posteromedial nucleus, ventral posterolateral nucleus [VPL], and ventral posterior nucleus parvicellular part). In experiments Thal2–5 there was also variable filling of the medial thalamus. There was some spread of tracer into regions surrounding PoT (e.g., medial geniculate nucleus [MG] and anterior pretectal nucleus [APT]) in each experiment, together with variable extension into the basal ganglia. There was no spread of tracer into the hypothalamus.

Injections targeted on the PoT (Fig. 2) filled all or most of this region in seven experiments (PoT3–9), whereas tracer filled only the rostral half in PoT1 and the caudal part in PoT2; the injection in PoT10 was too far rostral and involved mainly the caudal part of the Po. There was invariably spread into nearby structures such as: Po, MG, and APT, as well as suprageniculate, lateral posterior, and posterior intralaminar (PIL) thalamic nuclei. In experiment PoT2 there was significant spread of tracer into the midbrain but not into the PAG. In several cases (PoT1, 3–6, and 10) there was some rostral spread of tracer into the VB complex. There was no spread of tracer into the hypothalamus in any of these experiments.

In the double injection experiments, Fluoro-Gold was targeted on thalamic nuclei rostral to interaural 4.0 mm, and these were partially (DI1–4) or almost completely (DI5–7) filled (Fig. 3). Fluoro-Gold extended caudally to involve rostral PoT in DI4, and into caudal Po in DI1, 3, and 7. PoT was partially or completely filled with CTb in all experiments. Neither tracer spread into the hypothalamus in any of these experiments.

### Quantitative analysis of lamina I spinothalamic tract neurons in C7 and L4

**Figure 5:**
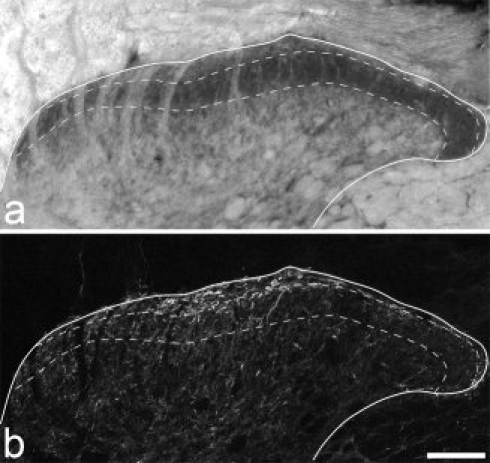
Identification of lamina I in transverse sections of C7. **a:** Darkfield photomicrograph of the C7 segment from experiment Thal5. The continuous white line outlines the gray matter, and the two dashed lines show the limits of the dark band that corresponds to lamina II. Note that lamina I is wider in the central part of the dorsal horn than in the lateral or medial parts. **b:** The same section scanned to reveal NK1r-immunoreactivity. The plexus of strongly immunoreactive profiles that occupies lamina I is also wider in the central part of the dorsal horn, and its dorsoventral extent closely matches the region defined as lamina I with darkfield microscopy. The confocal image in b is a projection of six optical sections at 4-μm z-spacing. Scale bar = 100 μm in b (applies to a,b).

The width of lamina I in the C7 segment (as judged by darkfield microscopy) was somewhat greater in the central region (up to 35 μm) than in the lateral or medial parts, where it was generally between 10 and 15 μm thick (Fig. 5a). An identical pattern was seen with the plexus of NK1r-immunoreactive dendrites and cell bodies that occupies lamina I (Fig. 5b).

The distributions of retrogradely labeled cells were generally similar across all of the experiments in which CTb or Fluoro-Gold were used as tracers. In the C7 segment, moderate numbers of retrogradely labeled neurons were observed in each section, and these were found in lamina I and scattered throughout the deeper laminae (III–VI) of the dorsal horn and the ventral horn. Occasional labeled cells were seen in lamina II. Although the distribution of retrogradely labeled cells in laminae I–V was similar in the L4 segment, these cells were far less numerous, for example, most transverse 60-μmVibratome sections contained no labeled lamina I neurons.

**Figure 6:**
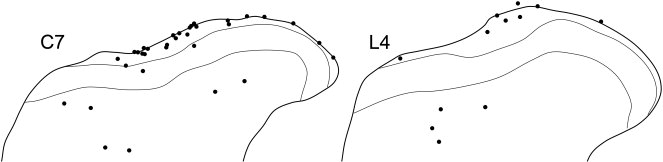
Plots of the locations of spinothalamic tract neurons at cervical and lumbar levels. These drawings show the location of all retrogradely labeled cells identified in the upper part of the dorsal horn in the transverse sections that were used for quantitative analysis in experiment Thal1. Each symbol represents a single neuron. The two thin lines indicate the dorsal and ventral borders of lamina II, which were determined from darkfield micrographs. The drawing on the left shows the cells seen in 10 randomly selected 60-μm sections through the C7 segment, whereas the drawing on the right represents the cells in 20 such sections from the L4 segment.

**Figure 7:**
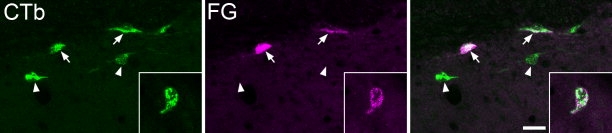
Retrograde labeling of lamina I spinothalamic tract neurons with CTb and Fluoro-Gold. The main part of the figure shows part of lamina I in a transverse section from the C7 segment of experiment DI3 immunostained to reveal CTb (green) and Fluoro-Gold (FG; magenta). A merged image is shown on the right. Several retrogradely labeled lamina I cells are present in this field. Two of these (arrows) contain both CTb and Fluoro-Gold, and others (two of which are indicated with arrowheads) are labeled with CTb but not Fluoro-Gold. The inset shows a single lamina I neuron in a transverse section from the L4 segment of experiment DI5 that was labeled with both CTb and Fluoro-Gold. The images were obtained from a projection of 19 (main part) or 18 (inset) confocal optical sections at 1-μm z-spacing. Scale bar = 20 μm in far right panel (applies to all).

Quantitative data for retrogradely labeled lamina I neurons are shown in Tables 2–4. The locations of retrogradely labeled cells in the upper part of the dorsal horn that were sampled in a representative experiment are illustrated in Figure 6, and examples of labeled lamina I cells are shown in Figure 7. The mean number of lamina I cells per 600 μm in the C7 segment was 23.2 for experiments Thal1–5 and 24.6 for experiments PoT1–9. (Data from PoT10 were not included in this part of the analysis, because the injection did not spread into PoT.) The corresponding numbers per 600 μm for the L4 segment were 3.5 for Thal1–5 and 3.6 for PoT1–9 (Tables 2, 3). In experiments DI1–7, the mean numbers of cells that were retrogradely labeled (with either tracer) per 600 μm in the C7 and L4 segments were 22.4 and 4.6, respectively (Table 4). Comparison of these values with those obtained in experiments Thal1–5 and PoT1–9 indicated that there were no significant differences between the numbers of retrogradely labeled cells found in each series for either cervical or lumbar segments (one-way ANOVA, *P* > 0.05 for C7 and L4).

In all of the double injection experiments apart from DI6, the majority of retrogradely labeled lamina I cells in the C7 segment contained CTb. In the L4 segment the majority of labeled lamina I cells contained CTb except in experiments DI6 and 7 (Table 4).

**Figure 8:**
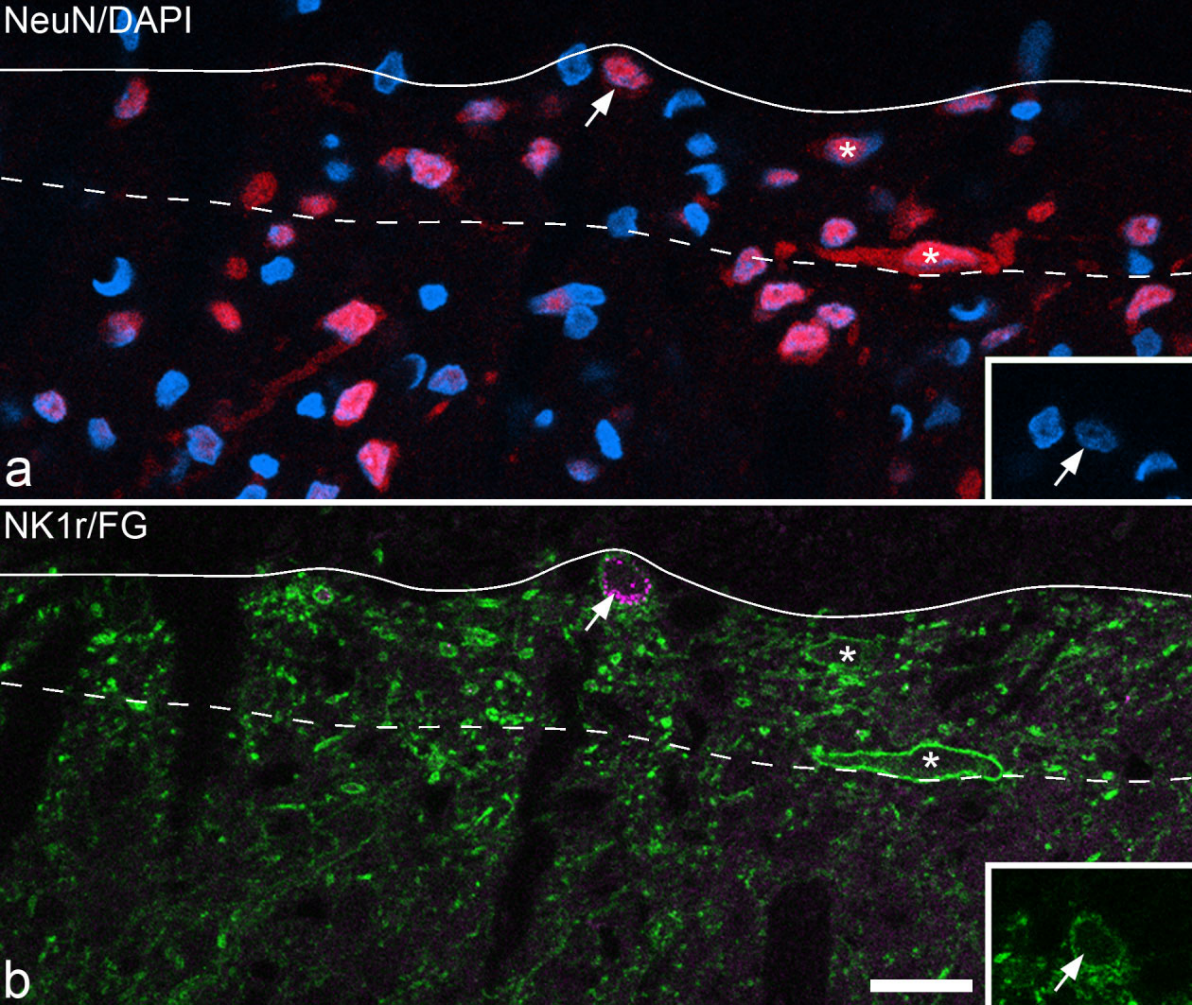
A lamina I spinothalamic neuron with NK1r. **a:** A transverse section through the medial part of lamina I in the C7 segment of experiment Thal3 scanned to reveal NeuN (red) and DAPI (blue). Neuronal nuclei contain both NeuN and DAPI and therefore appear magenta, whereas nonneuronal nuclei are blue. **b:** The same field scanned to reveal NK1r (green) and Fluoro-Gold (FG; magenta). The continuous and dashed lines show the upper and lower borders of lamina I, respectively. The arrow indicates a retrogradely labeled neuron in lamina I, which is also NK1r-immunoreactive. Two other NK1r-immunoreactive neurons that are not retrogradely labeled are shown with asterisks, and several neurons that are NK1r-negative and lack Fluoro-Gold are also present in lamina I. The upper inset shows DAPI staining in the nucleus of the spinothalamic neuron, and the lower inset shows the NK1r on its surface membrane. All images are projections of two optical sections at 1-μm z-spacing. Scale bar = 20 μm in b (applies to a,b).

For experiments Thal 2-5, PoT1, and PoT4-10, the retrogradely labeled lamina I neurons were examined for the presence of NK1r immunoreactivity. When data from these experiments were pooled, it was found that 255 out of 294 (87%) of the cells in C7 and 65 out of 77 (84%) of those in L4 were NK1r immunoreactive (Fig. 8).

In the sections of C7 that were used to determine the proportion of lamina I neurons belonging to the spinothalamic tract, numerous neuronal (NeuN-positive) profiles with DAPI-labeled nuclei were visible in lamina I, and many of these were also NK1r-immunoreactive (Fig. 8). Although Fluoro-Gold-positive cells were seen in lamina I in most sections, these were greatly outnumbered by neurons that were not retrogradely labeled. The total numbers of lamina I neurons in the disector samples in each experiment varied from 139 to 168 (mean 150, n = 4), whereas the numbers that were retrogradely labeled in these samples ranged from 1 to 7 (mean 3.5). The mean percentage of the sampled lamina I neurons that were retrogradely labeled was 2.4% (range 0.6–5%). Estimates of the total number of lamina I neurons per 600 μm length of C7 (calculated according to the formula in Materials and Methods) for the four experiments ranged from 1,028 to 1,420 (mean 1,254). The mean number of retrogradely labeled lamina I neurons determined for all PoT, Thal, and DI experiments (excluding PoT10; see above) was 23.5 per 600 μm (data from Tables 2–4, and this corresponds to 1.9% of the estimated total population of lamina I neurons.

The mean length of the C7 segment was 2.3 mm (range 2.2–2.4, n = 3).

### Proportion of lamina III/IV NK1r-immunoreactive neurons that project to thalamus

**Figure 9:**
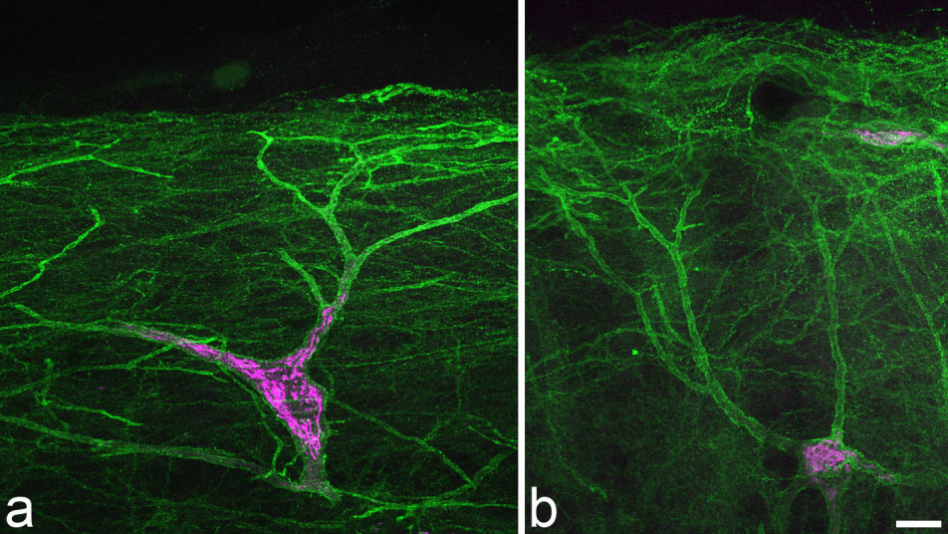
Lamina III NK1r-expressing spinothalamic neurons in parasagittal sections. **a:** A retrogradely labeled neuron in the C6 segment from experiment PoT7; **b:** A retrogradely labeled neuron in L5 from experiment DI6. In each case, CTb is shown in magenta and NK1r in green, and the dorsal limit of the dorsal horn is near the top of the field. Note that both of the labeled neurons have extensive dorsal dendrites that pass through the superficial dorsal horn. Images built from projections of 36 (a) or 60 (b) confocal optical sections at 1-μm z-spacing. Scale bar = 20 μm in b (applies to a,b).

As reported previously (Brown et al., 1995; Littlewood et al., 1995) we observed large NK1r-immunoreactive neurons in laminae III and IV in parasagittal sections of the L5 segment, and these often had dendrites that could be followed into the superficial dorsal horn (laminae I–II) (Fig. 9b). Similar cells were observed in the C6 segment (Fig. 9a), although these were somewhat less numerous than those in L5 (Tables 5–7. When data from Thal, PoT, and DI experiments were pooled, the mean number of NK1r-immunoreactive cells with somata in laminae III–IV and dendrites that could be followed into the superficial dorsal horn was 22.1 on the right side in the L5 segment and 15.8 on the right side in C6. The cells were uniformly distributed along both the mediolateral and rostrocaudal extent of the dorsal horn in each segment. The cell bodies were located between 93 and 405 μm (mean 223 μm ± 63, n = 419 cells) below the dorsal white matter in L5 and between 82 and 349 μm (mean 161 μm ± 42, n = 316 cells) below the dorsal white matter in C6.

In the C6 segment, most cells of this type were retrogradely labeled in all of the Thal, PoT, and DI experiments (except for PoT10; Tables 5–7. Comparison of the percentage values obtained in each group indicated that there were no significant differences between them (one-way ANOVA, *P* > 0.05). The overall proportion labeled was 85% (data pooled for all experiments apart from PoT10). In all of the DI experiments except for DI4, all retrogradely labeled cells of this type contained CTb, whereas double-labeled cells were only observed in DI1, 4, and 5 (Table 7).

In contrast, in the L5 segment the majority of the cells of this type were not retrogradely labeled in any of the Thal, PoT, or DI experiments (Tables 5–7. Comparison of the percentage values obtained in each group indicated that there were no significant differences between them (one-way ANOVA, *P* > 0.05). The overall proportion labeled was 17% (data pooled for all experiments apart from PoT10). In the DI experiments, all of the retrogradely labeled cells of this type in L5 contained CTb, and some of those in DI1, 4, and 6 were double-labeled (Table 7).

Results of the analysis of mediolateral distribution of retrogradely labeled lamina III/IV neurons are shown in Tables 5–7. When data from experiments Thal1–5, PoT3–9, DI1–4, and DI6–7 were pooled, the mean percentage of neurons in the medial group that were retrogradely labeled was 27, whereas for the lateral group the mean percentage was 7. These values were significantly different (*P* < 0.001, t-test).

### Retrograde labeling with fluorescent latex microspheres

**Figure 10:**
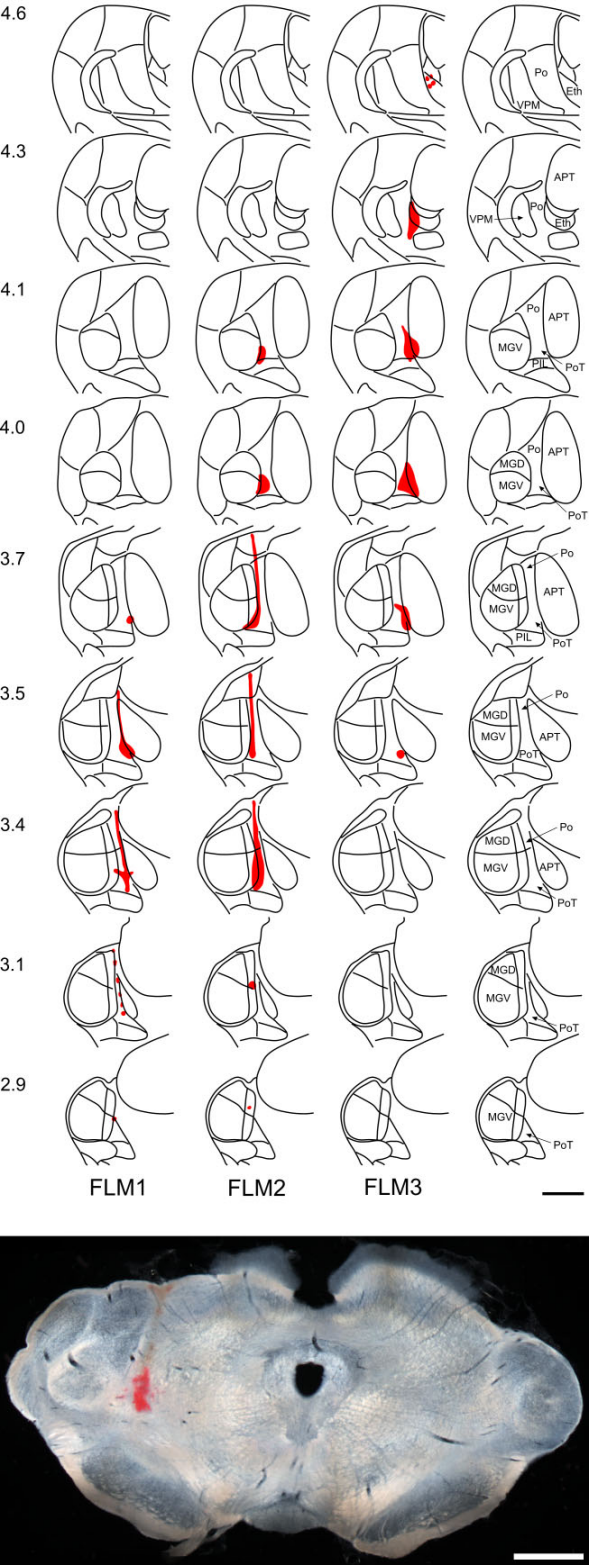
Injection sites for experiments FLM1-FLM3. Drawings show the spread of fluorescent latex microspheres (red) in these experiments. Each column represents a single experiment, and the experiment number (corresponding to those in Tables 1 and 8) is shown at the bottom of the column. Numbers at the top left of each drawing give the approximate position of the section anterior to the interaural plane. Drawings are based on those in Paxinos and Watson (2005). Eth, ethmoid thalamic nucleus; MGD, medial geniculate nucleus, dorsal part; MGV, medial geniculate nucleus, ventral part; other abbreviations as in Figure 1. The image below shows a darkfield photomicrograph (interaural ∼3.9 mm) from experiment FLM3. Note the limited spread of the red fluorescent latex microspheres. Scale bar = 1 mm in top and bottom panels.

Figure 10 shows drawings of the spread of fluorescent latex microspheres in experiments FLM1–3 and an example of an injection site from FLM3. In all cases, the spread of tracer was very limited, compared with that seen with CTb or Fluoro-Gold.

**Figure 11:**
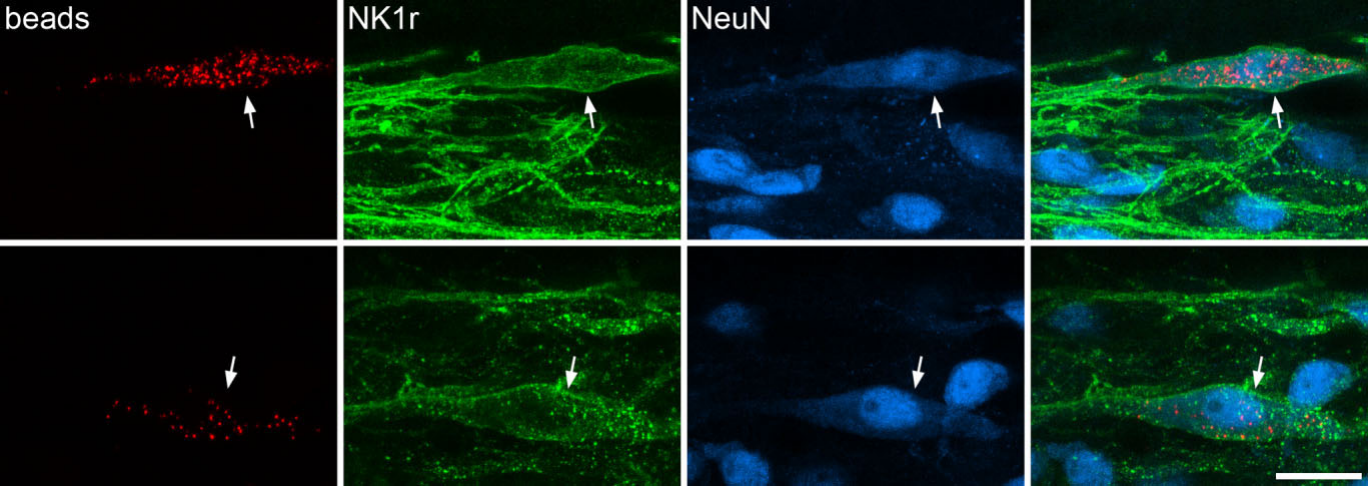
Retrograde labeling of lamina I neurons with fluorescent latex microspheres in parasagittal sections. The top row of images shows an example of a labeled lamina I neuron from C6 in experiment FLM3, and the bottom row shows a labeled neuron in L5 from experiment FLM2. In each case, separate images show the fluorescent latex microspheres (beads, red), NK1r (green) and NeuN (blue), with a merged image on the right. Note the presence of numerous beads in each neuron and that both neurons are NK1r-immunoreactive (arrow). Images are projections of seven (top row) or five (bottom row) confocal optical sections at 1-μm z-spacing. Scale bar = 20 μm in bottom right panel (applies to all).

**Figure 12:**
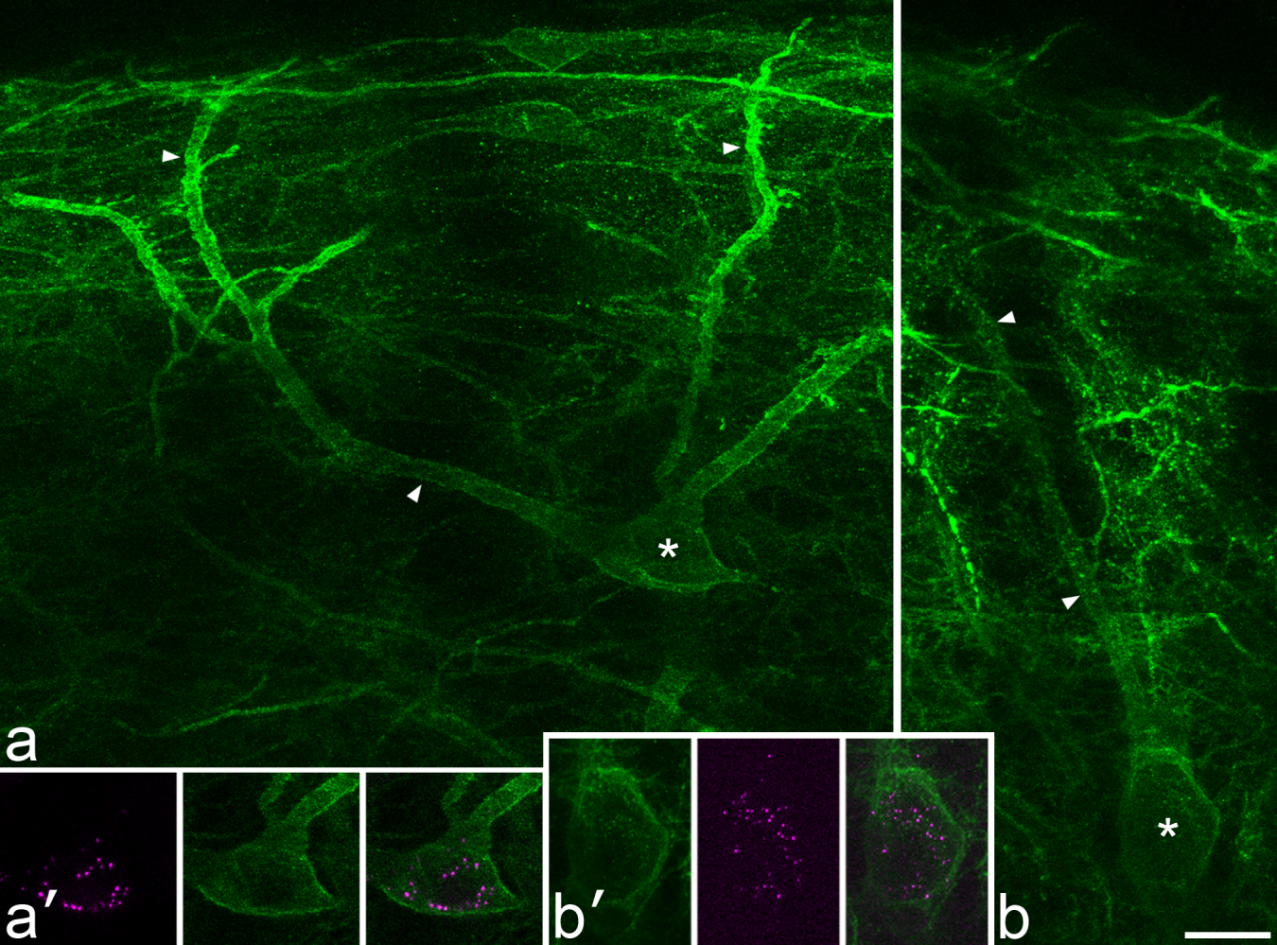
Retrograde labeling of NK1r-expressing neurons in lamina III with fluorescent latex microspheres in parasagittal sections. **a,b:** NK1r-immunoreactive lamina III cells in C6 (a) and L5 (b) from experiment FLM3. In each case, the soma is marked with an asterisk, and dorsal dendrites (arrowheads) can be seen passing into the superficial dorsal horn. **a′, b′:** The region of the soma of each cell scanned to reveal fluorescent latex microspheres (magenta) and NK1r (green), together with a merged image. Note the presence of numerous microspheres in the soma of each cell. a is a projection of 14 confocal optical sections at 1-μm z-spacing, and b is a montage of two fields projected from 14 and 9 optical sections at the same spacing. a′ and b′ are projections of 6 and 11 optical sections at 1-μm z-spacing, respectively. Scale bar = 20 μm in b (applies to a,b).

Examples of retrogradely labeled neurons are shown in Figures 11 and 12, and quantitative data are provided in Table 8. The numbers of microspheres in labeled neurons varied considerably: only cells that contained 5 or more were considered to be retrogradely labeled, and most such cells contained more than 10 microspheres. The optical properties of Gel-Mount were not as good as those of Vectashield, and although fluorescent latex microspherescould be identified through the depth of the section, it was difficult to follow NK1r-immunoreactive profiles deep within the sections. In the C6 segment, lamina I was found to contain between 15 and 46 retrogradely labeled cells, whereas 5–10 retrogradely labeled lamina III/IV NK1r-immunoreactive cells were observed, of which 1–6 had dendrites that could be followed into the superficial dorsal horn (Fig. 12a). Because of the difficulty in following NK1r-immunoreactive dendrites in these sections, only those cells that had dorsal dendrites that entered lamina II in the superficial part of the same section could be unequivocally identified as belonging to this type. In the L5 segment, no retrogradely labeled cells were detected in laminae I or III/IV in experiment FLM1, whereas in experiments FLM 2 and FLM3, 4 and 5 lamina I cells (respectively) and 4 and 6 lamina III/IV NK1r cells (respectively) were seen. Dorsal dendrites of two of the lamina III/IV NK1r-immunoreactive cells in experiment FLM3 could be followed into the superficial dorsal horn, and one of these is illustrated in Figure 12b. Although many of the retrogradely labeled lamina I cells in C6 and L5 could be identified as NK1r-immunoreactive (Fig. 11), when the cell was in the deepest part of the section, it was not possible to assess NK1r immunoreactivity, and therefore the proportion of cells that expressed the receptor was not quantified.

**Table 8:** Quantitative Data From FLM Experiments^1^

	Lamina I		Lamina III/IV NK1r-immunoreactive	
		
	C6	L5	C6	L5
FLM1	15	0	5 (1)	0
FLM2	37	4	10 (6)	4 (0)
FLM3	46	5	7 (3)	6 (2)

1 Counts of retrogradely labeled neurons in cervical and lumbar segments from experiments in which injections of fluorescent latex microspheres (FLM) were targeted on the PoT. Complete series of parasagittal sections through the dorsal horn of C6 and L5 segments were examined, and the total number of retrogradely labeled cells was counted. For the lamina III/IV NK1r-immunoreactive cells, the number of retrogradely labeled neurons for which dorsal dendrites could be followed into lamina II is given in parentheses (see text for further details).

## DISCUSSION

The major findings of this study are: 1) that injections of CTb or Fluoro-Gold targeted on PoT can label a high proportion of lamina I spinothalamic neurons at lumbar and cervical levels, 2) that ∼17% of lamina III/IV NK1r-immunoreactive neurons with long dorsal dendrites in L5 and ∼85% of those in C6 belong to the spinothalamic tract, and 3) that the lamina III/IV cells are not retrogradely labeled by injections of Fluoro-Gold restricted to the VB complex, rostral part of Po, and medial thalamus.

### Thalamic projection targets of lamina I neurons

Retrograde (Granum, 1986; Lima and Coimbra, 1988; Burstein et al., 1990; Marshall et al., 1996; Li et al., 1996; Yu et al., 2005) and anterograde (Peschanski et al., 1983; LeDoux et al., 1987; Cliffer et al., 1991) tracing studies, together with early degeneration studies (Lund and Webster, 1967; Zemlan et al., 1978), have indicated that the rat spinothalamic tract originates from several parts of the spinal gray matter and terminates widely in the thalamus in regions that include VPL, Po, mediodorsal, centrolateral, parafascicular, and ventromedial nuclei. In some reports, specific populations of spinothalamic neurons were identified (Giesler et al., 1979; Dado and Giesler, 1990; Kobayashi, 1998), and these demonstrated that the lamina I component projects mainly to lateral thalamus. A recent anterograde study confirmed that neurons in cervical superficial dorsal horn project to Po and VPL and also demonstrated a substantial projection to the caudal end of the thalamus (Gauriau and Bernard, 2004a). This latter projection extended from interaural ∼3.2–4.4 mm and was centred on PoT.

Because PoT may not have been included in injection sites in previous studies, our first aim was to determine whether injections that included this region would label a larger number of lamina I spinothalamic neurons than previously reported. In the Thal series, injections included all thalamic regions known to receive significant input from superficial dorsal horn, including PoT (Gauriau and Bernard, 2004a). Although there was some variation between injections, numbers of retrogradely labeled lamina I neurons in C7 were very consistent, averaging 23.2 cells per 600 μm. The mean number in L4 was considerably lower: 3.5 cells per 600 μm. Because the length of C7 was 2.3 mm, whereas that of L4 is ∼2.5 mm (Polgár et al. 2004), we estimate that there are ∼90 lamina I spinothalamic neurons on the contralateral side in C7 and 15 in L4. It is unlikely that this difference results from the longer transport distance from lumbar segments, because numerous lamina I neurons in the lumbar enlargement were labeled within a 3-day survival period following injections of CTb or Fluoro-Gold into CVLM, LPb, or PAG (e.g., Spike et al., 2003).

Two previous studies have quantified spinothalamic neurons in the rat: Lima and Coimbra (1988) estimated that there were 24 lamina I spinothalamic cells per side in C7 and 38 in L3–4, whereas Burstein et al. (1990) reported that there were 110 such cells in C7–8 and 19 in L4–5. Our estimates are considerably higher than those in either of these studies for the C7 segment, but not for L4. The injections in these two previous studies did not apparently fill PoT consistently, which suggests that injecting tracer into PoT increases the number of labeled lamina I spinothalamic cells in the cervical, but not lumbar enlargement.

Surprisingly, the mean numbers of retrogradely labeled neurons in the PoT experiments were very similar to those for the Thal series. Although there was some spread of tracer into surrounding structures in these experiments, this did not involve areas known to receive input from the superficial dorsal horn, except as an extension of the terminal field centered on PoT (Gauriau and Bernard, 2004a). This suggests that injections targeted on PoT can label most (if not all) spinothalamic neurons in lamina I.

If the difference between numbers of labeled lamina I neurons in C7 in the present study and those reported previously is due to inclusion of PoT in our injections, this suggests that some lamina I cells in this segment project to PoT but no further rostrally. This was explored in the DI experiments (CTb injection into PoT, Fluoro-Gold injection into more rostral thalamic nuclei). If this interpretation was correct, we would expect to find that most lamina I cells in C7 were double-labeled or only contained CTb. The total numbers of spinothalamic lamina I cells in the DI experiments were similar to those in Thal and PoT experiments, whereas the mean number labeled with Fluoro-Gold in C7 was lower (Table 4). However, there was considerable variation in the numbers of Fluoro-Gold- and CTb-labeled cells in this series, and cells labeled only with Fluoro-Gold were seen in significant numbers in some experiments. This may be explained partly by incomplete filling of PoT with CTb in some cases and may also result from the closeness of the Fluoro-Gold injection site to PoT resulting in suppression of CTb uptake, as it has been reported that injecting Fluoro-Gold proximal to other tracers in double-labeling experiments interferes with uptake of the second tracer (Akintunde and Buxton, 1992; Bice and Beal, 1997).

Although our results indicate that most lamina I spinothalamic neurons can be labeled by tracer injections into PoT, this might have resulted from uptake by fibers of passage, because the main bundle of spinothalamic axons passes through this region (LeDoux et al., 1987; Gauriau and Bernard, 2004a). Fluorescent latex microspheres are apparently not taken up by intact axons near the injection site (Pu and Amthor, 1990; Krug et al., 1998), and all three FLM experiments resulted in labeling of lamina I neurons in C6. In one experiment (FLM3) 46 of these cells were labeled and if the number of lamina I spinothalamic neurons in C6 is similar to that in C7, this would represent half of the spinothalamic population. Because the injection site in this animal occupied much less than half of the entire PoT, it is likely that this is a considerable underestimate. This suggests that most (if not all) lamina I spinothalamic neurons in the cervical enlargement have axons that arborize in or near PoT. Retrogradely labeled lamina I neurons were also seen in L5 in two of these experiments, and although the numbers were small, they represent approximately one-third of the expected population at this level. This indicates that some lamina I spinothalamic cells from the lumbar enlargement also project to PoT.

Taken together with previous reports, our findings suggest that the great majority of lamina I spinothalamic neurons have axons that pass through or close to PoT. Some of these continue rostrally to terminate in VPL and Po (in many cases giving off collaterals to PoT), whereas others terminate in PoT. This is consistent with a recent study by Zhang and Giesler (2005), who reported that a significant number of lamina I spinothalamic cells in rat cervical enlargement projected to PoT and surrounding regions, but no further rostrally.

### The proportion of lamina I neurons that project to the thalamus and their NK1r expression

The two methods that we used to estimate the proportion of lamina I neurons in C7 that belong to the spinothalamic tract gave slightly different values (2.4% and 1.9%). However, these are both much lower than the estimate by Yu et al. (2005), who concluded that 9% of lamina I neurons in the cervical enlargement were spinothalamic. It is difficult to explain the discrepancy between these results, although one significant methodological difference is the definition of the lamina I/II border in the two studies. We estimated the position of this border from the NK1r plexus whereas Yu et al. assumed a uniform width of 20 μm for lamina I. We therefore recalculated the percentages based on the assumption that lamina I had a width of 20 μm. Although this gave slightly higher values (3.2% and 2.1%), these are still far below that of Yu et al. (2005), and the method of placing the lamina I/II border therefore cannot account for these differences.

We did not directly determine the proportion of lamina I neurons in L4 that project to the thalamus, but this can be estimated from previously published data. Spike et al. (2003) used a similar disector method and concluded that there were 8,318 lamina I neurons per side in L4. However, in that study correction for tissue shrinkage was not performed, and this will therefore be an overestimate. In the present study the mean thickness of sections when scanned with the confocal microscope was found to be 45 μm, corresponding to a shrinkage of 25%. The tissue used by Spike et al. was prepared in the same way and is therefore likely to have undergone similar shrinkage. If this correction is applied retrospectively to the data from Spike et al., the number of lamina I neurons per side in L4 would be 6,238. Because we found 15 spinothalamic lamina I cells in L4, these would constitute 0.2% of the neuronal population in this lamina.

Marshall et al. (1996) reported that 77% of lamina I spinothalamic neurons in lumbar cord were NK1r-immunoreactive, and ∼80% of lumbar lamina I neurons labeled from CVLM, LPb, or PAG express the receptor (Todd et al., 2000). Here, we obtained a similar result for spinothalamic lamina I neurons in L4 (84%) and C7 (87%). NK1r-immunoreactive lamina I projection neurons in lumbar cord receive strong monosynaptic input from substance P-containing (nociceptive) primary afferents (Todd et al., 2002), and the NK1r-expressing spinothalamic lamina I neurons presumably provide a powerful input from these afferents to the thalamus.

### NK1r-expressing spinothalamic neurons in laminae III–IV

Lamina III/IV NK1r-immunoreactive neurons with long dorsal dendrites in lumbar cord also receive many synapses from substance P-containing afferents (Naim et al., 1997) and respond to various types of noxious stimulus (Polgár et al., 2007). Most of these cells can be labeled from CVLM and around two-thirds project to LPb (Todd et al., 2000). The present results show that ∼17% of those in L5 project to the thalamus, and this proportion is higher for cells located medially. Because the medial dorsal horn receives inputs from distal regions of the hindlimb (Swett and Woolf, 1985), distal limb inputs presumably have preferential access to the thalamus through spinothalamic cells of this type. Similar NK1r-immunoreactive cells have been identified in cervical cord (Brown et al., 1995), and we have found that these also receive numerous contacts from substance P-containing afferents (Al-Khater and Todd, unpublished observations). Here we show that the great majority of cells of this type in C6 belong to the spinothalamic tract.

In the DI experiments, very few of these cells in either C6 or L5 were labeled with Fluoro-Gold except in two cases (DI1 and 4) in which the Fluoro-Gold spread into rostral PoT. The other experiments in this series had extensive thalamic filling extending rostrally from interaural 4.6 mm, and including the VB complex, Po, and medial thalamus. The lack of Fluoro-Gold labeling in these experiments suggests that whereas these cells project to PoT, their axons do not arborize elsewhere in the thalamus. Zhang and Giesler (2005) identified spinothalamic neurons in laminae III–IV of rat cervical enlargement that projected only to caudal thalamus (mainly PoT and PIL), and their sample may have included some NK1r-expressing cells with long dorsal dendrites.

PoT projects to several areas, including S2 and insular cortices (Shi and Cassell, 1998; Linke and Schwegler, 2000), regions activated by painful stimuli in human imaging studies (Coghill et al., 1994; Casey et al., 1996). Gauriau and Bernard (2004b) demonstrated that rat PoT contains nociceptive-specific neurons that project to S2, as well as cells activated by noxious and tactile stimuli that project to insular cortex and amygdala. Most lamina I neurons are activated by noxious stimuli (Christensen and Perl, 1970; Bester et al., 2000), and these are likely to provide inputs to nociceptive-specific cells in PoT. Lamina III/IV NK1r-immunoreactive neurons receive monosynaptic inputs from myelinated (presumed low-threshold mechanoreceptive) primary afferents in lamina III, although these are much less numerous than those from substance P-containing (nociceptive) afferents (Naim et al., 1997,^1998^). These cells may therefore have wide dynamic range receptive fields and provide input to those PoT neurons that project to insular cortex and amygdala (Gauriau and Bernard, 2004b). Because lamina III/IV NK1r-immunoreactive neurons do not appear to project significantly to the VB complex, it is unlikely that their activity is transmitted directly to the primary somatosensory cortex. This is consistent with the small size of this population of cells, which means that they are unlikely to provide accurate information about stimulus localization, an important function of the primary somatosensory cortex.
